# Biological and Spectral Studies of Newly Synthesized Triazole Schiff Bases and Their Si(IV), Sn(IV) Complexes

**DOI:** 10.1155/2011/654250

**Published:** 2011-08-04

**Authors:** Kiran Singh, Parvesh Puri, Yogender Kumar, Chetan Sharma, Kamal Rai Aneja

**Affiliations:** ^1^Department of Chemistry, Kurukshetra University Kurukshetra 136 119, India; ^2^Department of Microbiology, Kurukshetra University Kurukshetra 136 119, India

## Abstract

The Schiff bases HL^1-3^ have been prepared by the reaction of 5-bromothiophene-2-carboxaldehyde with 4-amino-5-mercapto-3-methyl/propyl/isopropyl-s-triazole, respectively. Organosilicon(IV) and organotin(IV) complexes of formulae (CH_3_)_2_MCl(L^1-3^), (CH_3_)_2_M(L^1-3^)_2_ were synthesized from the reaction of (CH_3_)_2_MCl_2_ and the Schiff bases in 1 : 1 and 1 : 2 molar ratio, where M = Si and Sn. The synthesized Schiff bases and their metal complexes have been characterized with the aid of various physicochemical techniques like elemental analyses, molar conductance, UV, IR, ^1^H, ^13^C, ^29^Si, and ^119^Sn NMR spectroscopy. Based on these studies, the trigonal bipyramidal and octahedral geometries have been proposed for these complexes. The ligands and their metal complexes have been screened *in vitro* against some bacteria and fungi.

## 1. Introduction

Recently, the research relating with metal complexes of heteronuclear Schiff bases has expanded enormously and now comprising their interesting aspects in coordination chemistry with a special emphasis in bioinorganic chemistry. A use of organosilicon and organotin compounds as reagents or intermediates in the inorganic synthesis has further strengthened their applications [1, 2].

More-over, metal complexes of organosilicon(IV) and organotin(IV) halides with N, O, and S donor ligands have received much more consideration due to their industrial, environmental, and biological applications [3–5]. The N, O and S donor ligands have been used to enhance the biological activity of organosilicon and organotin derivatives [6]. Organosilicon(IV) complexes have been subjected of interest for their versatile applications in pharmaceutical and chemical industries. Organosilicon compounds of nitrogen and sulphur containing ligands are well known for their anticarcinogenic, antibacterial, antifungal, tuberculostatic, insecticidal, and acaricidal activities [7–10]. Generally, organosilicon complexes seem to owe their antitumor properties to the immune-defensive system of the organism [11]. Similarly, organotin compounds are the active components in a number of biocidal formulations in such diverse areas as fungicides, miticides, molluscicides, antifouling paints and surface disinfectants [12, 13]. In addition, many organotin compounds have been tested for a large variety of tumor lines and found to be more effective than traditional heavy metal anticancer drugs [14, 15]. Ahmad et al. have also screened some organotin compounds against tumor cells [16]. Prompted by these applications, few new organosilicon and organotin compounds have already been synthesized and screened for antibacterial and antifungal activities [17, 18], and in continuation to this, in the present paper, the synthesis, characterization, and biological activities of new triazole Schiff bases and their organosilicon and organotin complexes have been carried out.

## 2. Experimental

Dried solvents were used for the synthesis of compounds. Reagents,  5-bromothiophene-2-carboxaldehyde (Spectrochem), Dimethylsilicon-dichloride (Acros) and Dimethyltindichloride (TCI-America) were used as such.

### 2.1. Analytical Methods and Physical Measurements

Silicon and tin were determined gravimetrically as silicondioxide (SiO_2_) and tindioxide (SnO_2_). Melting points were determined on a capillary melting point apparatus. Molar conductance measurements of 10^−3^ M solution of metal complexes in dry DMF were measured at room temperature (25 ± 1°C) with a conductivity bridge type 305 Systronic model. Carbon, hydrogen, nitrogen and sulfur were estimated using elemental analyzer Heraeus Vario EL-III Carlo Erba 1108 at CDRI Lucknow. The electronic spectra of the ligands and their metal complexes were recorded in dry methanol, on a Systronics, Double-beam spectrophotometer 2203, in the range of 600–200 nm. The IR spectra of the ligands and metal complexes were recorded in nujol mulls/KBr pellets using BUCK scientific M5000 grating spectrophotometer in the range of 4000–350 cm^−1^. Nuclear magnetic resonance spectra (^1^H, ^13^C) were recorded on BRUKER-300ACF and ^29^Si and ^119^Sn were recorded on BRUKER-400ACF spectrometer in DMSO-d_6_ using tetramethylsilane (TMS) as an internal standard.

### 2.2. Synthesis of Ligands

**Figure 1:**
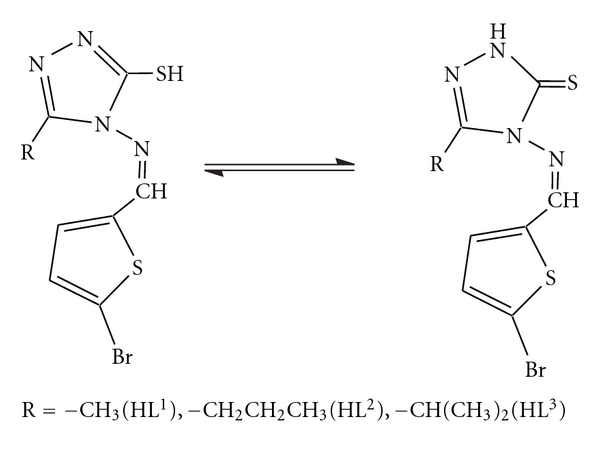
Structure of Schiff bases, where R = –CH_3_, HL^1^ = 4-(5-Bromothiophen-2-carboxylidene amino)-3-methyl-5-mercapto-s-triazole (BTMMT); R = –CH_2_–CH_2_–CH_3_, HL^2^ = 4-(5-Bromothiophen-2-carboxylidene amino)-5-mercapto-3-propyl-s-triazole (BTMPT); R = –CH(CH_3_)_2_, HL^3^ = 4-(5-Bromothiophen-2-carboxylidene amino)-3-isopropyl-5-mercapto-s-triazole  (BTIMT).

4-Amino-5-mercapto-3-methyl-s-triazole (AMMT), 4-amino-5-mercapto-3-propyl-s-triazole  (AMPT) and 4-amino-3-isoproyl-5-mercapto-s-triazole (AIMT) were synthesized by reported methods [19, 20]. The ligands were synthesized by condensation of 5-bromothiophene-2-carboxaldehyde with AMMT, AMPT and AIMT in the medium of ethanol (Figure 1). The contents were refluxed for 4–5 h in absolute ethanol. After refluxing, the reaction mixture was kept overnight at room temperature and the product was filtered, washed, and recrystallized from same solvent. The elemental analyses and physical properties of the ligands are reported in Table 1. The three ligands are: HL^1^ = 4-(5-Bromothiophen-2-carboxylidene amino)-3-methyl-5-mercapto-s-triazole (BTMMT), HL^2^ = 4-(5-Bromothiophen-2-carboxylidene amino)-5-mercapto-3-propyl-s-triazole (BTMPT), HL^3^ = 4-(5-Bromothiophen-2-carboxylidene amino)-3-isopropyl-5-mercapto-s-triazole (BTIMT).

### 2.3. Synthesis of Metal Complexes

To a weighed amount of dimethylsilicondichloride (Me_2_SiCl_2_) and dimethyltindichloride (Me_2_SnCl_2_) in ~30 mL of dry methanol, was added the calculated amount of the sodium salt of the ligands in 1 : 1 and 1 : 2 molar ratios. The sodium salts of the ligands were prepared by dissolving the appropriate amount of the sodium metal and ligands in ~30 mL dry methanol. The reaction mixture was refluxed for about 12 h and then allowed to cool at room temperature and removed the chlorine as sodium chloride. The excess of solvent was removed under reduced pressure by vacuum pump and the resulting solid was repeatedly washed with 5–10 mL dry cyclohexane and again dried under vacuum. The elemental analyses and physical properties of the complexes are reported in Table 1.

## 3. Results and Discussion

The reactions of Me_2_SiCl_2_ and Me_2_SnCl_2_ with the sodium salt of monobasic bidentate ligands in 1 : 1 and 1 : 2 molar ratios in methanol medium result in the precipitation of sodium chloride (NaCl), as shown by following reactions:


(1)
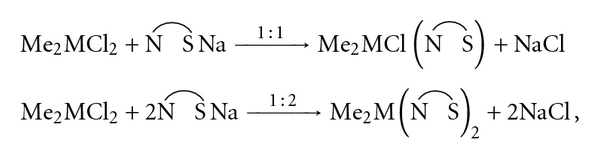

where M = Si or Sn and N S represent the donor sites of the ligands.

The resulting complexes have been obtained as coloured solids which are soluble in DMSO, DMF, and MeOH. The ligands show a sharp melting point, but the complexes decompose in a range of temperature (200–300°C). The molar conductivity values measured for 10^−3^ M solutions in anhydrous DMF are in the range of 10–16 Ω^−1^cm^2^ mol^−1^, showing that all 1 : 1 and 1 : 2 complexes are nonelectrolytic in nature Table 1. 

### 3.1. Electronic Spectra

The electronic spectra of the ligands HL^1−3^ and their corresponding Si(IV) and Sn(IV) metal complexes were recorded. The electronic spectra of ligands HL^1^, HL^2^, and HL^3^ exhibit maxima at 388 nm, 364 nm, and 387 nm, respectively, which could be assigned to the n-π* transition of the azomethine group. These bands show a blue shift in 1 : 1 and 1 : 2, Si(IV) and Sn(IV) metal complexes and appear at 368 nm, 369 nm, 358 nm, 369 nm, 362 nm, 364 nm, 359 nm, 362 nm, 372 nm, 368 nm, 376 nm and 366 nm for Me_2_SiCl(L^1^), Me_2_Si(L^1^)_2_, Me_2_SnCl(L^1^), Me_2_Sn(L^1^)_2_, Me_2_SiCl(L^2^), Me_2_Si(L^2^)_2_, Me_2_SnCl(L^2^), Me_2_Sn(L^2^)_2_, Me_2_SiCl(L^3^), Me_2_Si(L^3^)_2_, Me_2_SnCl(L^3^), and Me_2_Sn(L^3^)_2_, respectively, and indicating the coordination of azomethine nitrogen atom to the metal atom [16]. In addition to this, the three medium intensity bands at 244 nm, 240 nm, and 260 nm due to *π*-π* transition in the ligands remain unchanged or show a minor change in the spectra of metal complexes [17].

### 3.2. IR Spectra

In the IR spectra of the ligands, a broad band in the region of 3117–3094 cm^−1^ due to *ν*(N–H) [13] and a band at ~1120 cm^−1^ due to *ν*(C=S) [21], indicating the thione form, while a weak band observed around 2750 cm^−1^ due to *ν*(S–H) vibrations suggested that the Schiff bases exhibit thiol-thione tautomerism (Figure 1) [22, 23]. The deprotonation of −SH group of triazole was indicated by the absence of bands in the spectra of metal complexes due to *ν*(S–H), *ν*(C=S), and *ν*(N–H). A new band appears ~740 cm^−1^ in the spectra of the complexes, which is assigned to *ν*(C–S) and which indicates the complexation of ligands through S-atom with the metal atom. The metal sulphur bond formation is further supported by a band at ~452 cm^−1^ and ~426 cm^−1^ for *ν*(Si–S) [24] and *ν*(Sn–S) [25] in the spectra of organosilicon and organotin complexes, respectively. A sharp and strong band in the region of 1582–1597 cm^−1^ for *ν*(N=CH) [26] in case of ligands, was shifted to a higher wavelength number and appears in the region of 1628–1674 cm^−1^ in the spectra of metal complexes, indicating the coordination of ligands through azomethine nitrogen to the metal atom. The metal nitrogen bond was further supported by the presence of a band at about ~535 cm^−1^ for *ν*(Sn–N) [27] and ~575 cm^−1^ for *ν* (Si–N) [28]. A strong band in the region of 425–378 cm^−1^ was assigned to *ν*(M–Cl) [29]. The IR-spectral data of the ligands and their metal complexes are listed in Table 2.

### 3.3. ^1^H NMR Spectra

**Figure 2:**
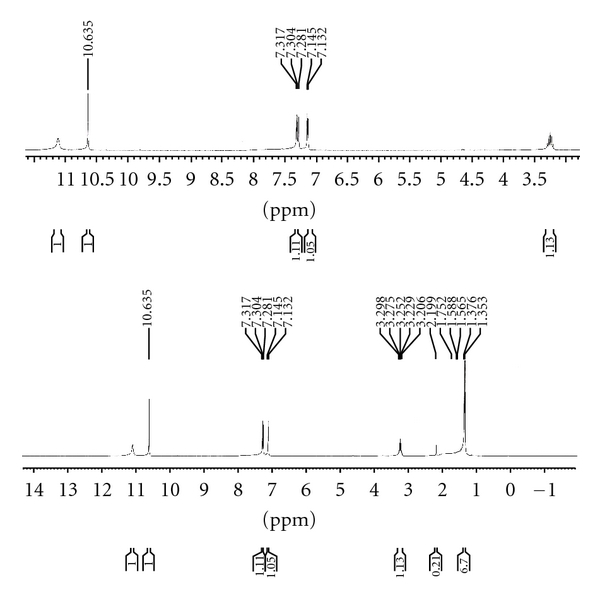
^1^H NMR spectrum of Schiff base (HL^3^).

**Figure 3:**
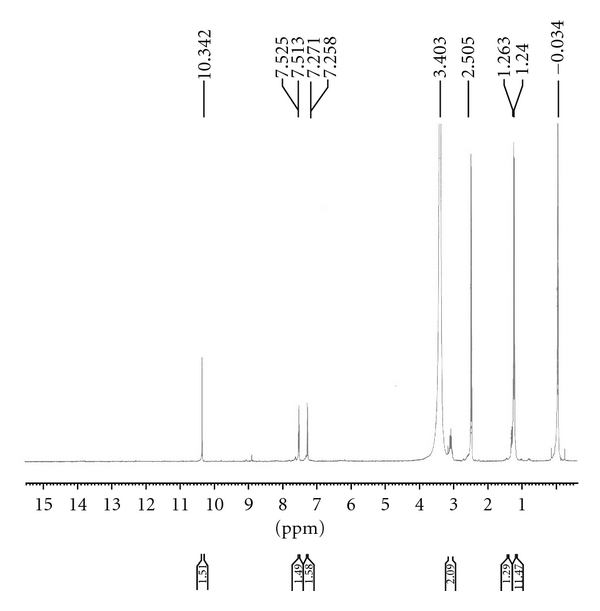
^1^H NMR spectrum of Si (1 : 1) metal complex of ligand (HL^3^).

The ^1^H NMR spectra of the ligands show the –SH proton signal at *δ* 10.47 (s), *δ* 13.75 (s), and *δ* 11.10 (s) ppm for HL^1^, HL^2^, and HL^3^, respectively [26] (Figure 2). The disappearance of the signal due to –SH proton in the spectra of metal complexes indicates the deprotonation of the thiol group and supports the coordination of ligand through sulphur atom to the metal atom. A signal at *δ* 11.72 (s), 10.91 (s), and 10.63 (s) ppm was observed due to azomethine proton in the spectra of free ligands HL^1^, HL^2^ and HL^3^, respectively, which moves upfield in the ^1^H NMR spectra of metal complexes [13], indicates the bonding through the azomethine nitrogen atom to the central metal atom (Figure 3). The aromatic protons of the thiophene moiety in the ligands appear as two doublets, which remain more or less unchanged in the ^1^H NMR spectra of the metal complexes. Some additional signals at *δ* 2.45 ppm (s, C**H_3_**, Triazole), *δ* 2.78 ppm (t, C**H_2_**–CH_2_–CH_3_, Triazole), *δ* 1.69–1.63 ppm (m, CH_2_–C**H_2_**–CH_3_, Triazole), *δ* 1.03 ppm (t, CH_2_–CH_2_–C**H_3_**, Triazole), *δ* 3.29–3.20 ppm (C**H**(CH_3_)_2_, Triazole), *δ* 1.36 ppm (d, CH(C**H_3_**)_2_, Triazole) and also appeared in the ^1^HNMR spectra of the ligands, and their metal complexes, reported in the Table 3. The additional signals in the region *δ* 0.3–1.5 ppm are also observed in the spectra of complexes due to CH_3_–M group.

### 3.4. ^13^C NMR Spectra

The ^13^C NMR spectral data of ligands HL^1^, HL^2^, and HL^3^, and their corresponding 1 : 1 and 1 : 2 metal complexes [17, 18] have been reported in Table 4. The signal due to the carbon atom attached to the azomethine group in the ligands HL^1^, HL^2^, and HL^3^ appear at *δ* 166.42 ppm, *δ* 162.23 ppm, and *δ* 160.79 ppm, respectively. However, in the spectra of the corresponding metal complexes, the shift in the ^13^C resonance indicate the coordination of nitrogen atom of azomethine group with the central atom in 1 : 1 and 1 : 2 metal complexes. Moreover, the shifting of the ^13^C resonance of triazole which is attached to sulphur atom in the spectra of 1 : 1 and 1 : 2 metal complexes compared to the free ligands indicates the coordination through sulphur atom with the central metal atom. The new signal due to the methyl groups attached to the metal atom in the spectra of metal complexes has also been reported in Table 4.

### 3.5. ^29^Si and ^119^Sn NMR Spectra

**Figure 4:**
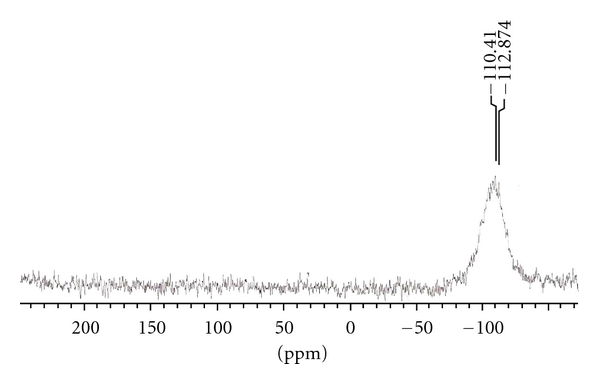
^29^Si NMR spectrum of Si (1 : 1) metal complex of ligand (HL^1^).

The value of *δ* ^29^Si and *δ* ^119^Sn indicates the coordination number of the central metal atom in the corresponding complexes [30], and generally (Figure 4), ^29^Si and ^119^Sn chemical shifts move to lower frequency with increasing coordination number of the metal atoms. The spectrum shows in each case only a sharp singlet indicating the formation of single species. ^29^Si and ^119^Sn NMR spectra of {Me_2_SiCl(L^1^)},{Me_2_Si(L^1^)_2_},{Me_2_SnCl(L^1^)}, and {Me_2_Sn(L^1^)_2_} complexes show sharp signals at *δ*−110.41 ppm, *δ*−123.35 ppm, *δ*−176.46 ppm, and *δ*−265.26 ppm, respectively, Which is indicative of pentacoordinated and hexacoordinated around the silicon and tin atom [8].

## 4. Biological Activities

The bactericidal and fungicidal activities of the free ligands and their metal complexes against various gram positive and gram negative bacteria and fungi are reported in Tables 5, 6, and 7.

### 4.1. In Vitro Antibacterial Assay

The newly synthesized ligands and their metal complexes were screened for their antibacterial activities against test bacteria namely *Staphylococcus aureus, Bacillus subtilis *(Gram positive), *Escherichia coli, *and *Pseudomonas aeruginosa *(Gram negative). The activity is determined by reported Agar well diffusion method [31, 32]. All the microbial cultures were adjusted to 0.5 McFarland standards, which is visually comparable to a microbial suspension of approximately 1.5 × 10^8^ cfu/mL. 20 mL of Mueller Hinton Agar medium was poured into each petri plate and plates were swabbed with 100 *μ*L inocula of the test microorganisms and kept for 15 min for adsorption. Using sterile cork borer of 8 mm diameter, wells were bored into the seeded agar plates, and these were loaded with a 100 *μ*L volume with concentration of 4.0 mg/mL of each compound reconstituted in the DMSO. All the plates were incubated at 37°C for 24 hrs. Antibacterial activity of each synthetic compound was evaluated by measuring the zone of growth inhibition against the test organisms with zone reader (Hi Antibiotic zone scale). DMSO was used as a negative control, whereas Ciprofloxacin was used as positive control. This procedure was performed in three replicate plates for each organism.

### 4.2. Determination of Minimum Inhibitory Concentration (MIC)

MIC of the various compounds against bacterial strains was tested through a macrodilution tube method as recommended by NCCLS [33]. In this method, the various test concentrations of synthesized compounds were made from 128 to 0.25 *μ*g/mL in sterile tubes nos. 1 to 10. 100 *μ*L sterile Mueller Hinton Broth (MHB) was poured in each sterile tube followed by addition of 200 *μ*L test compound in tube 1. Twofold serial dilutions were carried out from tube 1 to the tube 10 and excess broth (100 *μ*L) was discarded from the test tube no. 10. To each tube, 100 *μ*L of standard inoculum (1.5 × 10^8^ cfu/mL) was added. Ciprofloxacin was used as control. Turbidity was observed after incubating the inoculated tubes at 37°C for 24 hrs.

### 4.3. In Vitro Antifungal Activity

The ligands and their metal complexes were also screened for their antifungal activity against two fungi, namely, *A. niger *and* A. flavus*, the ear pathogens isolated from the patients of Kurukshetra [34], by poison food technique [35]. The moulds were grown on Sabouraud dextrose agar (SDA) at 25°C for 7 days and used as inocula. The 15 mL of molten SDA (45°C) was poisoned by the addition of 100 *μ*L volume of each compound having concentration of 4.0 mg/mL reconstituted in the DMSO, poured into a sterile petri plate and allowed it to solidify at room temperature. The solidified poisoned agar plates were inoculated at the center with fungal plugs (8 mm diameter) obtained from the colony margins and incubated at 25°C for 7 days. DMSO was used as the negative control whereas Fluconazole was used as the positive control. The experiments were performed in triplicates. Diameter of fungal colonies was measured and expressed as percent mycelial inhibition by applying the formula.

(2) $$\text{Percent}\;\text{inhibition}\;\text{of}\;\text{mycelial}\;\text{growth}=\frac{dc-dt}{dc\times 100},$$

where *dc* is the average diameter of fungal colony in negative control sets and *dt* is the average diameter fungal colony in experimental sets.

### 4.4. Observations

**Figure 5:**
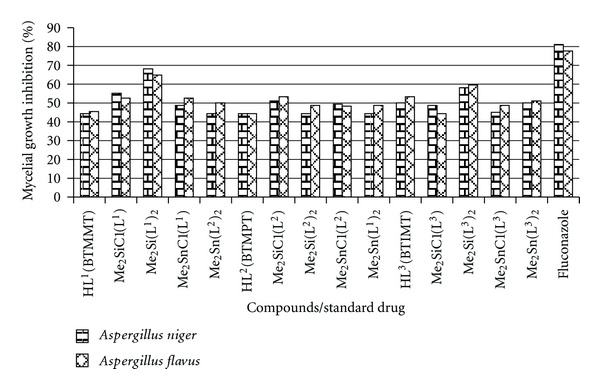
Comparison of antifungal activity of compounds with commercial antibiotic.

The antibacterial data reveals that the complexes are superior compared to the free ligands. The free ligands and their metal complexes are active against Gram-positive bacteria (*Staphylococcus aureus *and* Bacillus subtilis)* and inactive against gram negative bacteria (*Escherichia coli *and *Pseudomonas aeruginosa*). Among the synthesized compounds tested compounds, Me_2_SnCl(L^1^) and Me_2_SiCl(L^2^) show more antibacterial activity that is, near to standard drug (Ciprofloxacin) (Table 5). In the series, the MIC of the compounds ranged between 28–128 *μ*g/mL against Gram-positive bacteria. Compound Me_2_SnCl(L^1^) and Me_2_SiCl(L^2^) show highest MIC of 28 *μ*g/mL against *S. aureus* (Table 6). The antifungal activity of compounds (Figure 5) shows more than 50% inhibition of mycelia growth against *Aspergillus niger *and* A. flavus* (Table 7). Thus, it can be postulated that further studies of these complexes in this direction could lead to more interesting results.

## 5. Conclusion

**Figure 6:**
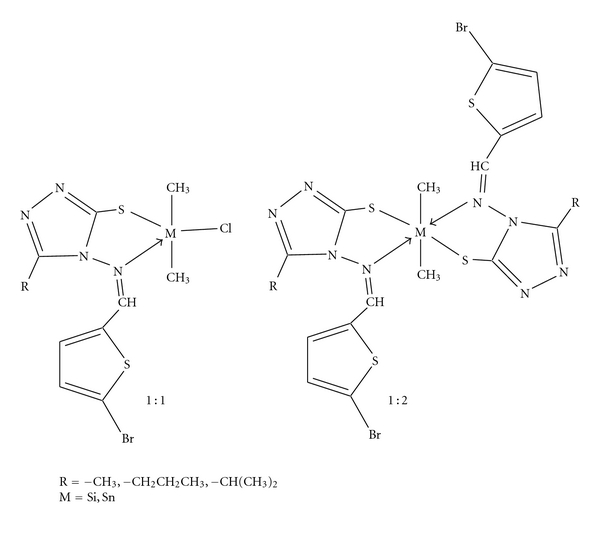
Proposed structures of the 1 : 1 and 1 : 2 complexes, where 1 : 1 complexes, coordination number = 5 are proposed to have trigonal bipyramidal and 1 : 2 complexes, coordination number = 6 are proposed to have octahedral geometries.

Trigonal bipyramidal and octahedral geometries have been proposed for 1 : 1 and 1 : 2 organosilicon(IV) and organotin(IV) complexes with the help of various physico-chemical studies like IR, UV, ^1^H, ^13^C, ^29^Si, and ^119^Sn NMR (Figure 6). The free ligands, and their metal complexes were screened against various fungi and bacteria to access their potential as antimicrobial agents. The antimicrobial data reveals that the complexes are superior to the free ligands and their toxicity has increased as per the increase in concentration. These compounds were found more potent inhibitor of fungal growth as compared to the bacterial culture.

## Figures and Tables

**Table 1:** Physical characteristics and analytical data of ligands and their metal complexes.

Compound	Empirical formulae	Color	Decomposition Temp. (°C)	Molar conductance (Ω^−1^ cm^2^ mol^−1^)	Found (Calc.)%				
					C	H	N	S	Si/Sn
HL^1^(BTMMT)	C_8_H_7_BrN_4_S_2_	Light Brown	182	—	31.02 (31.69)	2.43 (2.33)	18.64 (18.48)	21.21 (21.15)	—
Me_2_SiCl(L^1^)	C_10_H_12_BrClN_4_S_2_Si	Brown	176	15.46	30.04 (29.44)	3.21 (3.05)	14.43 (14.24)	16.12 (16.20)	7.12 (7.10)
Me_2_Si(L^1^)_2_	C_18_H_18_Br_2_N_8_S_4_Si	Light Yellow	220	11.24	32.21 (32.63)	2.87 (2.74)	16.78 (16.91)	19.32 (19.36)	4.25 (4.24)
Me_2_SnCl(L^1^)	C_10_H_12_BrClN_4_S_2_Sn	Yellow	222	14.82	24.98 (24.69)	2.65 (2.49)	11.44 (11.52)	13.19 (13.18)	24.35 (24.40)
Me_2_Sn(L^1^)_2_	C_18_H_18_Br_2_N_8_S_4_Sn	Light Yellow	238	10.78	28.64 (28.70)	2.44 (2.41)	14.34 (14.88)	17.06 (17.03)	15.74 (15.76)
HL^2^(BTMPT)	C_10_H_11_BrN_4_S_2_	Dark Brown	178	—	36.84 (36.26)	3.66 (3.35)	16.79 (16.91)	19.42 (19.36)	—
Me_2_SiCl(L^2^)	C_12_H_16_BrClN_4_S_2_Si	Brown	172	15.98	34.42 (34.00)	3.54 (3.80)	13.42 (13.22)	15.21 (15.13)	6.67 (6.63)
Me_2_Si(L^2^)_2_	C_22_H_26_Br_2_N_8_S_4_Si	White	234	11.43	36.21 (36.77)	3.55 (3.65)	15.61 (15.59)	17.57 (17.85)	3.89 (3.91)
Me_2_SnCl(L^2^)	C_12_H_16_BrClN_4_S_2_Sn	White	224	14.52	28.76 (28.01)	3.24 (3.13)	10.02 (10.08)	12.51 (12.47)	23.10 (23.07)
Me_2_Sn(L^2^)_2_	C_22_H_26_Br_2_N_8_S_4_Sn	White	260	10.54	32.42 (32.65)	3.12 (3.24)	13.58 (13.85)	15.81 (15.85)	14.65 (14.67)
HL^3^(BTIMT)	C_10_H_11_BrN_4_S_2_	Light Brown	174	—	36.44 (36.26)	3.36 (3.35)	16.96 (16.91)	19.38 (19.36)	—
Me_2_SiCl(L^3^)	C_12_H_16_BrClN_4_S_2_Si	Pale Yellow	244	15.88	34.06 (34.00)	3.70 (3.80)	13.44 (13.22)	15.18 (15.13)	6.67 (6.63)
Me_2_Si(L^3^)_2_	C_22_H_26_Br_2_N_8_S_4_Si	Light Yellow	252	11.47	36.44 (36.77)	3.46 (3.65)	15.62 (15.59)	17.79 (17.85)	3.89 (3.91)
Me_2_SnCl(L^3^)	C_12_H_16_BrClN_4_S_2_Sn	Dark Brown	262	13.49	28.12 (28.01)	3.42 (3.13)	10.10 (10.08)	12.42 (12.47)	23.10 (23.07)
Me_2_Sn(L^3^)_2_	C_22_H_26_Br_2_N_8_S_4_Sn	Light Yellow	272	10.21	32.42 (32.65)	3.27 (3.24)	13.72 (13.85)	15.91 (15.85)	14.69 (14.67)

**Table 2:** IR-spectroscopic data (cm^−1^) of the ligands and their metal complexes.

Compound	*ν*(N–H)	*ν*(–C=N)	*ν*(C=S)^a^/*ν*(C–S)^b^	*ν*(S–H)	*ν*(M–S)	*ν*(M–N)	*ν*(M–Cl)
HL^1^(BTMMT)	3117	1597	1173	2754	—	—	—
Me_2_SiCl(L^1^)	—	1628	717	—	453	572	418
Me_2_Si(L^1^)_2_	—	1628	710	—	458	576	—
Me_2_SnCl(L^1^)	—	1643	741	—	403	528	378
Me_2_Sn(L^1^)_2_	—	1643	741	—	416	538	—
HL^2^(BTMPT)	3109	1589	1111	2754	—	—	—
Me_2_SiCl(L^2^)	—	1636	702	—	452	570	420
Me_2_Si(L^2^)_2_	—	1697	741	—	446	582	—
Me_2_SnCl(L^2^)	—	1674	741	—	416	542	396
Me_2_Sn(L^2^)_2_	—	1674	733	—	418	543	—
HL^3^(BTIMT)	3094	1582	1126	2777	—	—	—
Me_2_SiCl(L^3^)	—	1655	756	—	456	563	426
Me_2_Si(L^3^)_2_	—	1659	741	—	452	578	—
Me_2_SnCl(L^3^)	—	1651	764	—	410	536	395
Me_2_Sn(L^3^)_2_	—	1659	733	—	416	544	—

a = Ligands.

b = Complexes.

**Table 3:** ^1^HNMR chemical shifts of the ligands and their metal complexes.

Compound	–CH=N	–SH	Aromatic-H	Triazole-CH_3_, –CH_2_–CH_2_–CH_3_, –CH(CH_3_)
HL^1^(BTMMT)	11.70 (s)	10.47 (s)	7.30 (d, 1H, *J* = 3.6 Hz); 7.13 (d, 1H, *J* = 3.6 Hz)	2.45 (s, 3H)
Me_2_SiCl(L^1^)	9.64 (s)	—	7.35 (d, 1H, *J* = 3.9 Hz); 7.14 (d, 1H, *J* = 3.9 Hz)	2.42 (s, 3H)
Me_2_Si(L^1^)_2_	11.12 (s)	—	7.42 (d, 2 H, *J* = 3.9 Hz); 7.31 (d, 2 H, *J* = 3.9 Hz)	2.19 (s, 6H)
Me_2_SnCl(L^1^)	11.19 (s)	—	7.26 (d,1H, *J* = 3.0 Hz); 7.14 (d, 1H, *J* = 3.0 Hz)	2.22 (s, 3H)
Me_2_Sn(L^1^)_2_	11.15 (s)	—	7.36 (d, 2H, *J* = 3.0 Hz); 7.29 (d, 2H, *J* = 3.0 Hz)	2.10 (s, 6H)
HL^2^(BTMPT)	10.91 (s)	13.75 (s)	7.31 (d, 1H, *J* = 3.6 Hz); 7.13 (d, 1H, *J* = 3.6 Hz)	2.78 (t, 2H, *J* = 7.5 Hz); 1.69–1.63 (m, 2H); 1.03 (t, 3H, *J* = 7.5 Hz)
Me_2_SiCl(L^2^)	10.41 (s)	—	7.44 (d, 1H, *J* = 3.9 Hz); 7.19 (d, 1H, *J* = 3.9 Hz)	2.64 (t, 2H, *J* = 7.5 Hz); 1.79–1.61(m, 2H); 0.94 (t, 3H, *J* = 7.5 Hz)
Me_2_Si(L^2^)_2_	8.41 (s)	—	7.43 (d, 2H, *J* = 3.9 Hz); 7.21 (d, 2H, *J* = 3.9 Hz)	2.63 (t, 4H, *J* = 7.5 Hz); 1.65–1.48 (m, 4H); 0.96 (t, 6H, *J* = 7.5 Hz)
Me_2_SnCl(L^2^)	8.49 (s)	—	7.20 (d, 1H, *J* = 3.9 Hz); 6.88 (d, 1H, *J* = 3.9 Hz)	2.62 (t, 2H, *J* = 7.5 Hz); 1.79–1.56 (m, 2H); 0.94 (t, 3H, *J* = 7.5 Hz)
Me_2_Sn(L^2^)_2_	8.87 (s)	—	7.36 (d, 2H, *J* = 3.6 Hz); 7.35 (d, 2H, *J* = 3.6 Hz)	2.68 (t, 4H, *J* = 7.2 Hz); 1.99–1.97 (m, 4H); 1.25 (t, 6H, *J* = 7.2 Hz)
HL^3^(BTIMT)	10.63 (s)	11.10 (s)	7.31(d, 1H, *J* = 3.9 Hz); 7.13 (d, 1H, *J* = 3.9 Hz)	3.29–3.20 (m, 1H); 1.36 (d, 6H, *J* = 6.9 Hz)
Me_2_SiCl(L^3^)	10.32 (s)	—	7.51 (d, 1H, *J* = 3.6 Hz); 7.23 (d, 1H, *J* = 3.6 Hz)	3.28–3.12 (m, 1H,); 1.25 (d, 6H, *J* = 7.2 Hz)
Me_2_Si(L^3^)_2_	8.44 (s)	—	7.10 (d, 2H, *J* = 3.9 Hz); 7.02 (d, 2H, *J* = 3.9 Hz)	3.14–2.86 (m, 2H); 1.25 (d,12H, *J* = 7.2 Hz)
Me_2_SnCl(L^3^)	8.40 (s)	—	7.12 (d, 1H, *J* = 3.9 Hz); 7.08 (d, 1H, *J* = 3.9 Hz)	2.87–2.73 (m, 1 H); 1.17 (d, 6 H, *J* = 7.2 Hz)
Me_2_Sn(L^3^)_2_	8.48 (s)	—	7.10 (d, 2H, *J* = 3.9 Hz); 7.09 (d, 2H, *J* = 3.9 Hz)	2.92–2.83 (m, 2H); 1.18 (d,12H, *J* = 7.2 Hz)

**Table 4:** C^13^ NMR chemical shifts of the ligands and their metal complexes.

Compound	C_1_	C_2_	C_3_	C_4_	C_5_	C_6_	C_7_	C_8_	C_9_	C_10_	M–CH_3_
HL^1^(BTMMT)	124.05	135.85	139.06	143.92	166.42	153.51	157.32	15.67	—	—	—
Me_2_SiCl(L^1^)	131.77	132.39	137.24	137.94	183.07	138.32	156.08	9.5	—	—	18.11
Me_2_Si(L^1^)_2_	116.70	132.06	133.60	141.23	160.96	147.79	149.04	11.32	—	—	28.22
Me_2_SnCl(L^1^)	120.65	132.03	132.98	142.52	162.54	147.89	148.26	11.65	—	—	30.11
Me_2_Sn(L^1^)_2_	116.03	131.94	132.82	141.82	161.23	147.33	147.58	11.37	—	—	32.11
HL^2^(BTMPT)	120.15	131.09	134.59	138.97	162.23	152.73	152.92	13.69	19.29	26.91	—
Me_2_SiCl(L^2^)	119.30	131.84	135.64	139.04	183.06	154.32	156.02	13.76	19.44	26.81	18.23
Me_2_Si(L^2^)_2_	128.18	129.28	131.98	141.72	161.81	153.26	154.25	14.58	19.02	25.95	24.66
Me_2_SnCl(L^2^)	126.23	128.56	131.23	140.58	162.48	152.56	154.85	14.23	18.65	26.42	31.32
Me_2_Sn(L^2^)_2_	124.26	130.45	132.05	141.62	161.98	153.26	154.26	14.42	18.87	26.86	32.00
HL^3^(BTIMT)	124.18	135.96	139.22	143.95	160.79	157.86	157.26	30.27	24.44	24.44	—
Me_2_SiCl(L^3^)	118.67	132.03	135.82	139.08	161.95	154.71	154.98	25.56	19.79	19.79	19.12
Me_2_Si(L^3^)_2_	120.42	129.45	133.25	138.55	160.78	152.53	151.25	28.45	20.25	22.76	29.10
Me_2_SnCl(L^3^)	122.62	128.46	132.46	139.42	161.86	153.24	154.25	27.56	19.45	24.57	29.88
Me_2_Sn(L^3^)_2_	124.56	130.54	135.03	141.21	162.46	154.48	153.24	28.89	21.22	26.43	31.89

**Table 5:** *In vitro* antibacterial activity of the ligands and their metal complexes.

Compounds	Zone of inhibition (mm)^a^			
	*S. aureus*	*B. subtilis*	*E. coli*	*P. aeruginosa*
HL^1^(BTMMT)	16.2	15.6	—	—
Me_2_SiCl(L^1^)	21.6	—	—	—
Me_2_Si(L^1^)_2_	20.3	—	—	—
Me_2_SnCl(L^1^)	24.6	22.6	—	—
Me_2_Sn(L^1^)_2_	—	18.6	—	—
HL^2^(BTMPT)	18.8	18.6	—	—
Me_2_SiCl(L^2^)	23.6	21.3	—	—
Me_2_Si(L^2^)_2_	17.3	15.2	—	—
Me_2_SnCl(L^2^)	—	—	—	—
Me_2_Sn(L^2^)_2_	15.3	16.3	—	—
HL^3^(BTIMT)	—	15.9	—	—
Me_2_SiCl(L^3^)	—	20.2	—	—
Me_2_Si(L^3^)_2_	—	16.2	—	—
Me_2_SnCl(L^3^)	—	16.8	—	—
Me_2_Sn(L^3^)_2_	—	15.3	—	—
Ciprofloxacin	27.6	26	—	—

—: No activity.

^a^Values, including diameter of the well (8 mm), are means of three replicates.

**Table 6:** Minimum inhibitory concentration (MIC) in *μ*g/mL of the ligands and their metal complexes.

Compound	*S. aureus*	*B. subtilis*
HL^1^(BTMMT)	>128	128
Me_2_SiCl(L^1^)	64	Nt
Me_2_Si(L^1^)_2_	64	Nt
Me_2_SnCl(L^1^)	28	54
Me_2_Sn(L^1^)_2_	Nt	64
HL^2^(BTMPT)	Nt	128
Me_2_SiCl(L^2^)	28	58
Me_2_Si(L^2^)_2_	128	>128
Me_2_SnCl(L^2^)	—	—
Me_2_Sn(L^2^)_2_	128	128
HL^3^(BTIMT)	Nt	128
Me_2_SiCl(L^3^)	Nt	128
Me_2_Si(L^3^)_2_	Nt	128
Me_2_SnCl(L^3^)	Nt	128
Me_2_Sn(L^3^)_2_	Nt	128
Ciprofloxacin	5	5

Nt: Not tested.

**Table 7:** *In vitro* antifungal activity of the ligands and their metal complexes.

Compound	Mycelial growth inhibition (%)	
	*Aspergillus niger*	*Aspergillus flavus*
HL^1^(BTMMT)	44.4	45.5
Me_2_SiCl(L^1^)	55.2	52.5
Me_2_Si(L^1^)_2_	**68.2**	**64.8**
Me_2_SnCl(L^1^)	48.8	52.5
Me_2_Sn(L^1^)_2_	44.4	50
HL^2^(BTMPT)	44.4	44.4
Me_2_SiCl(L^2^)	51.1	53.3
Me_2_Si(L^2^)_2_	44.4	48.8
Me_2_SnCl(L^2^)	49.4	48.4
Me_2_Sn(L^2^)_2_	44.4	48.8
HL^3^(BTIMT)	50	53.3
Me_2_SiCl(L^3^)	48.8	44.4
Me_2_Si(L^3^)_2_	**58.1**	**59.5**
Me_2_SnCl(L^3^)	45	48.8
Me_2_Sn(L^3^)_2_	50	51.1
Fluconazole	81.1	77.7
